# Using Hamming Distance as Information for SNP-Sets Clustering and Testing in Disease Association Studies

**DOI:** 10.1371/journal.pone.0135918

**Published:** 2015-08-24

**Authors:** Charlotte Wang, Wen-Hsin Kao, Chuhsing Kate Hsiao

**Affiliations:** 1 Institute of Epidemiology and Preventive Medicine, National Taiwan University, Taipei, 100, Taiwan; 2 Bioinformatics and Biostatistics Core, Division of Genomic Medicine, Research Center for Medical Excellence, National Taiwan University, Taipei, 100, Taiwan; 3 Department of Public Health, National Taiwan University, Taipei, 100, Taiwan; New Jersey Institute of Technology, UNITED STATES

## Abstract

The availability of high-throughput genomic data has led to several challenges in recent genetic association studies, including the large number of genetic variants that must be considered and the computational complexity in statistical analyses. Tackling these problems with a marker-set study such as SNP-set analysis can be an efficient solution. To construct SNP-sets, we first propose a clustering algorithm, which employs Hamming distance to measure the similarity between strings of SNP genotypes and evaluates whether the given SNPs or SNP-sets should be clustered. A dendrogram can then be constructed based on such distance measure, and the number of clusters can be determined. With the resulting SNP-sets, we next develop an association test HDAT to examine susceptibility to the disease of interest. This proposed test assesses, based on Hamming distance, whether the similarity between a diseased and a normal individual differs from the similarity between two individuals of the same disease status. In our proposed methodology, only genotype information is needed. No inference of haplotypes is required, and SNPs under consideration do not need to locate in nearby regions. The proposed clustering algorithm and association test are illustrated with applications and simulation studies. As compared with other existing methods, the clustering algorithm is faster and better at identifying sets containing SNPs exerting a similar effect. In addition, the simulation studies demonstrated that the proposed test works well for SNP-sets containing a large proportion of neutral SNPs. Furthermore, employing the clustering algorithm before testing a large set of data improves the knowledge in confining the genetic regions for susceptible genetic markers.

## Introduction

With the rapid advancements made in biotechnology, the volume and types of biological data collected have grown at an accelerated rate. Such an enormous amount of data complicates the analysis of genetic association studies, especially when dealing with complex diseases with single-marker tests, which are often hampered by the problems inherent in multiple testing and by low power [1,2]. To alleviate such problems, current approaches either change the unit of analysis to a group of markers and focus on reduction of data dimension, or select representative markers through several stages of analysis. The former philosophy of analysis is favored by many researchers, mostly because a set of markers may jointly contribute to an effect different from the additive effect of single markers. Existing multiple-marker analyses include candidate multiple-marker tests, haplotype association analysis, SNP-set analysis, and gene-set or pathway analysis [3–5].

Candidate multiple-marker tests and haplotype analysis are usually employed when the number of genetic markers is not large. For instance, Hotelling’s *T* ^2^ statistic and linear or logistic regression models with marker genotypes can include a moderately sized group of candidate markers in one model. These analyses are sensitive to genotype or allele frequencies, and work only for markers in the same, either protective or deleterious, direction [1]. As for haplotype analysis, it can directly take into consideration the possible interplay between individual markers, such as the linkage disequilibrium (LD) between SNPs. Three common measurements for LD are *D*, D′ and *r* ^2^. The first two compare the difference between haplotype and allelic frequencies; while the third quantity takes into account the allele frequencies in such differences. Such haplotype analyses, however, may not be straightforward because of the ambiguity in the determination of haplotype phase and the uncertainty in haplotype composition. In addition, computational burden becomes increasingly difficult as the number of markers located in the region of interest increases [6,7].

To explore the association between a wide genetic region and a disease, SNP-set analysis, gene set analysis, and pathway analysis are all more flexible approaches, in the sense that they are useful when analyzing a large pre-specified region based on investigators’ prior knowledge or any available biological information [8,9]. These tools therefore can model the joint effect of a group of variants that may be correlated with each other. These methods can be categorized as SNP-set analysis, because, in a broad sense, the unit of analysis in SNP-sets can be any arbitrary cluster of SNPs, located either in neighboring genomic regions or in genes from a known pathway [10].

To construct SNP-sets when no *a priori* knowledge is available, clustering algorithms may be utilized as an exploratory tool to integrate the information contained in a large number of SNPs. Unfortunately, most clustering algorithms are developed to summarize relationships among quantitative measurements. Only a few studies have discussed how to cluster discrete data like SNPs or to identify subsets of relevant SNPs [11,12]. These studies have applied similarity measures, such as matching coefficients, modified Pearson’s coefficients, or Spearman’s coefficients, to model the potential relationship between SNPs and epidemiological data. These clustering methods are applied separately on case and control groups and, thus, may result in different clusterings of SNP-sets for the two groups, leading to difficulties in interpretation. The measures such as *D*′ and *r* ^2^ for linkage disequilibrium can be utilized to group SNP variables as well. These are useful when haplotype information is available and when grouping loci in regions close to each other is of interest.

Another similarity measure for categorical variables is Hamming distance [13], a distance measure commonly employed in information theory to quantify similarity or dissimilarity among discrete data. This metric measures the dissimilarity between two strings of equal length, as a symmetric kernel between two vectors, and has been widely applied [14–16]. However, the properties of a clustering dendrogram constructed with the Hamming distance measure have not yet been investigated. Hamming distance calculates the number of dissimilar components between two strings so that all component-wise information is retained and no single marker needs to be removed. To determine if two groups of data should be clustered, Zhang et al. [16] tested if these two groups are equivalent based on the empirical distributions of Hamming distance. Their method was found to be useful as long as data were of a size moderate enough that convergence was be achieved in a reasonable amount of time. Another common algorithm, the k-mode, also tends to require a long running time and is sensitive to initial values [15,17]. In cases where clustering long regions of SNPs is of interest, the two issues of deciding when to merge SNP-sets and what procedure to use to compute distance remain critical.

For association tests, Hamming distance was previously considered as a symmetric kernel in tests proposed by Pinheiro et al. [18] and Wei et al. [19] in a test involving decomposition of the similarity measure between subjects. Pinheiro et al. [18] used the between-group component to define a test statistic and defined their Hamming distance as the proportion of discordant SNPs. Their test statistic is insensitive in detecting differences between groups since the value of the test statistic is often too small to achieve significance by bootstrap re-sampling. Wei et al. [19] used a weighted Hamming distance as the kernel and a ratio of a between-group statistic to a within-group statistic as the test quantity. They improved the nonparametric test proposed by Schaid et al. [20] by taking into account the dissimilarity between individuals from different treatment groups. Their between group statistic was the between group dissimilarity adjusted by a scalar of the within-group dissimilarity. This modification may not be compatible with heterogeneity between subjects of different traits, and thus its use may deserve some reservations. Furthermore, they considered the weight as the negative logarithm of the *p*-value from a single marker test, which may not be suitable when sample size is large or when minor allele frequency is small.

In regression models, other statistical tests based on different similarity measures have been proposed for use in association studies. Tzeng et al. [21,22] proposed a gene-trait similarity regression to detect associations between genetic variants and disease traits. They first derived a weighted cross product of trait residuals, and then regressed this product on genetic similarity. For a binary disease trait with no covariate information available, this is like comparing the average genetic similarity within a specific group, say the affected, with an average of the genetic similarity within the control group and the genetic similarity across groups. That is, the latter average considers both within-group and cross-group similarity. This latter average considers both the within and cross group similarity. Alternatively, Wessel and Schork [22] proposed regressing a genetic dis-similarity matrix of squared “distance” on a design matrix of phenotypes such as gene expressions to test for associations. They considered seven different distance metrics, including ibs and haplotype similarity, with various weights. For dichotomous phenotypes, this approach compares the average dissimilarity (the distance) within the affected group with the average dissimilarity within the normal group. It does not consider the cross-group dissimilarity. For case-control studies, the dissimilarity between a pair of individuals with different disease traits may exhibit larger deviation and thus reveal greater information about disease susceptibility, especially when evaluated on a causal set of markers. Therefore, such cross-group dissimilarity should be included in analysis, and should be considered separately from the within group dissimilarity.

The aim of our study is to develop a methodology of utilizing the Hamming distance metric to measure the distance between two sets of vectors containing discrete observations, in order to first perform clustering and then to use this clustering to conduct association studies. In the clustering stage, we modify the hierarchical clustering algorithm with average linkage for continuous data and develop a Hamming distance-based algorithm to determine SNP-sets. The procedure we develop is based on the rational that the more individuals carrying the same genotype with respect to two given SNPs (or two SNP-sets), the more similar these two SNPs (or sets) should be considered, which is exactly what the Hamming distance does by assigning them a smaller value. This Hamming distance dissimilarity measure can be considered as a function of ibs, or a special case of rare-allele-weighting similarity proposed by Wessel and Schork [23]. We also extend this idea to evaluate whether two SNP-sets should be considered close enough to be clustered together. In the second stage of our study, in which we investigate whether a given SNP-set obtained in the clustering stage is associated with the disease of interest, we propose a test statistic that compares the “distance” (or difference) between individuals with regard to their SNP profile for the given SNP-set. If the SNP-set is not associated with the disease, then the expected distance between a case and a normal subject should be the same as that between two individuals of the same disease status. Therefore, the focus of the second stage is on analyzing the difference between subjects in the disease and non-disease groups rather than the difference between SNPs. In contrast to the tests used by Pinheiro et al. [18] and Wei et al. [19], our proposed method does not depend on ANOVA decomposition and, instead, we simply compare the “distance” within groups and the “distance” across groups. The spirit of our test is similar to earlier tests in the use of similarity measures such as the “allele-match” kernel [18–23], but differs in its focus on comparing within-group and cross-group dissimilarity. Our statistic thus differs from those previously proposed. In addition, we place more emphasis on our statistic’s operational characteristics in a variety of settings. We also assess the performance of the proposed method under different settings of sample size, case-to-control ratio, and noise-to-signal ratio (the proportion of the number of causal SNPs to the number of non-disease-related SNPs). Applications are illustrated and simulation studies are conducted to evaluate the performance of the clustering algorithm and the association test.

## Methods

### Clustering SNP-sets with a Similarity Measure

**Fig 1:**
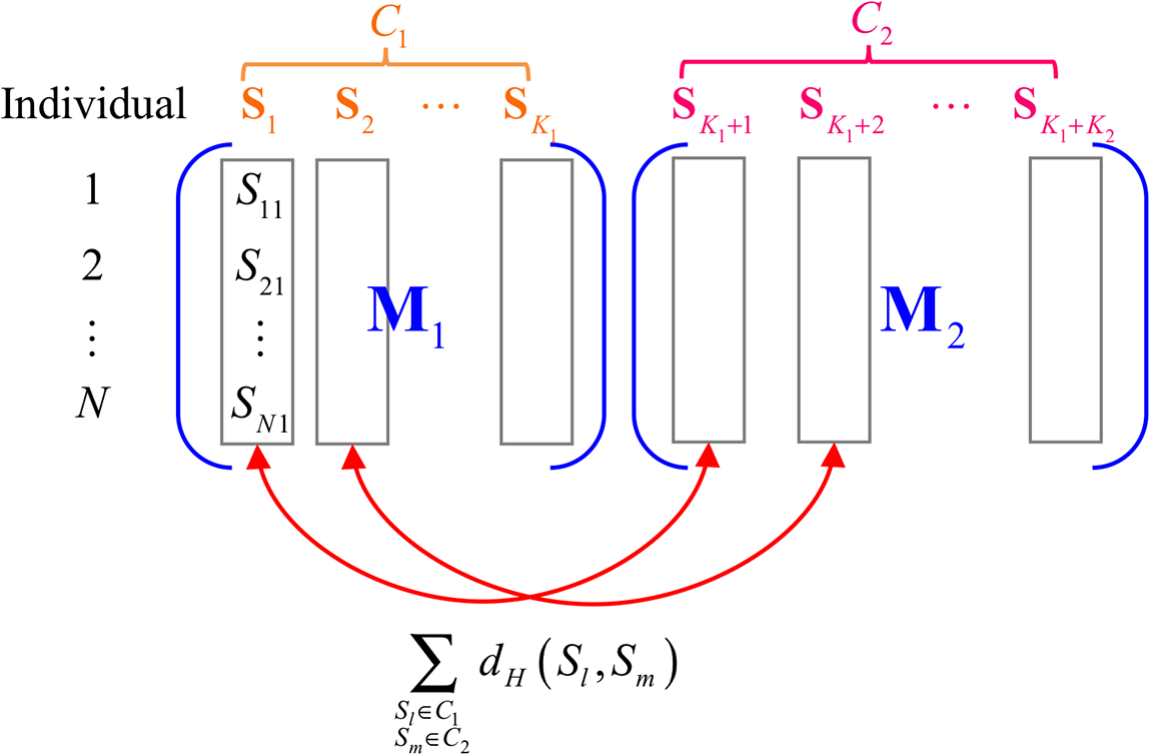
Schematic diagram of SNP clusters.

Let *C*_1_ and *C*_2_ be the two SNP-sets under study, where the sets contain *K*_1_ and *K*_2_ SNPs, respectively. For each SNP indexed *k*, the vector **S**_*k*_ denotes the genotypes of *N* individuals. In other words,

$$S_{k}={\left(S_{1k},S_{2k},\ldots ,S_{Nk}\right)}^{t}$$

is a SNP genotype vector of length *N*, where *S*_*ik*_ is the genotype of the *i*-th individual at the *k*th SNP and *k* = 1, 2, …, *K* (*K* = *K*_1_ + *K*_2_). All genotypes of the *K*_1_ SNPs in cluster *C*_1_ can be expressed in the form of an *N* × *K*_1_ matrix **M**_1_ with elements *S*_*ik*_. Similarly, the genotype matrix **M**_2_ for cluster *C*_2_ is of dimension *N* × *K*_2_. Fig 1 shows a schematic diagram of these two clusters.

#### Distance (dis-similarity) between SNP-sets

We define *d*(*C*_1_, *C*_2_) the dissimilarity between two SNP-sets *C*_1_ and *C*_2_ as the average dissimilarity of all possible pairs **S**_*l*_ and **S**_*m*_ from *C*_1_ and *C*_2_, respectively,

$$d(C_{1},C_{2})=\frac{1}{K_{1}\times K_{2}}\sum_{\begin{array}{l} S_{l}\in C_{1}\\ S_{m}\in C_{2} \end{array}}d_{H}(S_{l},S_{m}),$$

where *d*_*H*_(**S**_*l*_, **S**_*m*_) denotes the number of discordant components between two SNP strings, i.e. the number of individuals whose genotypes on SNP *l* and SNP *m* differ,

$$d_{H}(S_{l},S_{m})=\sum_{i=1}^{N}I(S_{il}\neq S_{im}).$$

The subscript *H* stands for Hamming distance. Note that some may prefer to compute the proportion, instead of the counts, of discordant components. These two definitions however are equivalent. This similarity measure is indicated in Fig 1 as well.

The quantity *d*(*C*_1_, *C*_2_) measures the average “dissimilarity” between all possible pairs of SNPs with one from each set. A large value indicates a greater degree of dissimilarity between these two sets, whereas a small value implies “resemblance” between two sets and may lead to an action of clustering. In matrix terminology, *d*(*C*_1_, *C*_2_) evaluates the average distance between a column **S**_*l*_ in **M**_1_ and a column **S**_*m*_ in **M**_2_.

#### Merging Procedure, Handling Ties and Dendrogram Construction

When there are *J* clusters, {*C*_1_, *C*_2_,…, *C*_*J*_}, the distances *d*(*C*_*i*_, *C*_*j*_) of all possible combinatorial selections of paired clusters ($C_{2}^{J}=J\times (J-1)/2$ pairs in this case) will be computed and the pair with the smallest distance will be merged into a new SNP-set, leading to *J*−1 clusters.

In case of a tie, the following rules are applied. If more than one pair shares the same minimum distance and these pairs involve non-overlapping sets, then each pair will be combined into a new set. When overlapping sets exist, the grouping strategy will depend on the way they overlap. For instance, if more than a pair from *C*_*A*_, *C*_*B*_ and *C*_*C*_ show the same minimum distance, then a random pair, say (*C*_*A*_, *C*_*B*_), will be combined, leaving *C*_*C*_ to be grouped in the next stage. However, if *C*_*A*_ has the same minimum distance with each of the other two distinct clusters *C*_*B*_ and *C*_*C*_, but *C*_*B*_ and *C*_*C*_ are not close to each other, then a union, either *C*_*A*_∪*C*_*B*_ or *C*_*A*_∪*C*_*C*_, will be selected at random to form a new cluster. This procedure guarantees that the number of clusters will be reduced by at least 1 in each stage. Therefore, if the clustering procedure starts with *K* SNPs, then this proposed procedure will proceed until all *K* SNPs are grouped into one final cluster to complete an agglomerative hierarchical clustering dendrogram.

#### Determination of Clusters

If *J* is the pre-specified number of clusters that investigators believe to exist for all SNPs under study, then these clusters can be identified by examining the dendrogram in a top-down order. When no knowledge about *J* is available, we trace for each SNP the clusters it visits sequentially and allocate the SNP to a cluster that shows the largest degree of dissimilarity with others. The steps of the procedure are as follows:

1. *Trace History of Clusters*: For each column vector SNP **S**_*k*_, trace all *n*_*k*_ clusters in the dendrogram that the SNP ever belonged to, and order these clusters from the smallest to largest $\left\{C_{k(1)},C_{k(2)},\ldots ,C_{k(n_{k})}\right\}$, where $C_{k(1)}\subset C_{k(2)}\subset \ldots \subset C_{k(n_{k})}$.
2. *Calculate Node Heights in Dendrogram*: Calculate for **S**_*k*_ the “height” *H*_*k*(*i*)_ of each node of its corresponding clusters,
  $$\begin{array}{l} H_{k(1)}=d(C_{k(1)},C_{k(2)}-C_{k(1)})\\ \vdots \\ H_{k(n_{k}-1)}=d(C_{k(n_{k}-1)},C_{k(n_{k})}-C_{k(n_{k}-1)}) \end{array}$$
  In the dendrogram, *H*_*k*(*i*)_ is the vertical length of a new node measured from the bottom, when the cluster *C*_*k*(*i*)_ becomes *C*_*k*(*i*+1)_. Therefore, $H_{k(1)}<H_{k(2)}<\cdots <H_{k(n_{k}-1)}$ for SNP **S**_*k*_. This quantity indeed measures the “distance” between two sets. Note that *C*_*k*(*i*+1)_ − *C*_*k*(*i*)_ denotes the difference in SNPs between sets *C*_*k*(*i*+1)_ and *C*_*k*(*i*)_, *C*_*k*(*i*+1)_ − *C*_*k*(*i*)_ = {**S**_*l*_: **S**_*l*_ ∈ *C*_*k*(*i*+1)_ and **S**_*l*_ ∉ *C*_*k*(*i*)_}. A toy example for computing *H*_*k*(*i*)_ is presented in S1 Text.
3. *Assign Cluster*: Compute the maximum “relative height” between successive nodes,

$$D(S_{k})=\underset{i=2,\ldots ,n_{k}-1}{\text{max}}\{H_{k(i)}-H_{k(i-1)}\},$$

where *H*_*k*(*i*)_ is the node height as defined above. This “relative height” is indeed the vertical distance between nodes. If *D*(**S**_*k*_) = *H*_*k*(*i*)_ − *H*_*k*(*i*−1)_ for some integer *i*, then *C*_*k*(*i*)_ is the cluster selected for SNP **S**_*k*_.

The result of the procedure is that every SNP will be assigned to a cluster where the size of each cluster may vary, and the number of clusters is now determined. The resulting clusters can then be used in the next stage, i.e. the association test. A hypothetical data set and dendrogram presented in additional file (S1 Text) illustrate calculation of the measures *H*_*k*(*i*)_ and *D*(**S**_*k*_).

If one prefers to work on a smaller number of clusters containing, for instance, only 5% of the SNPs for further investigation, then these SNPs and corresponding clusters can be retrieved by selecting SNPs whose corresponding *D*(**S**_*k*_) are greater than the 95% quantile of {*D*(**S**_1_), *D*(**S**_2_),…, *D*(**S**_*k*_)}. The resulting number of clusters, say *J*, for these SNPs can then be used. Alternatively, one can utilize gap statistics or similar quantities [24–26] to determine the number of clusters. These statistics have been developed to determine the optimal number of clusters from continuous observations [27].

### Association Tests with SNP-sets

Testing whether a SNP-set is associated with a disease of interest is equivalent to testing whether the set can distinguish different disease phenotypes. In other words, the unit of analysis now becomes the subjects, and the Hamming distance between paired subjects across all markers is to be evaluated. That is, the focus now is on the difference with respect to all markers between two paired individuals. If the SNP-set is independent of the disease, then any two individuals should carry similar markers in this SNP-set, regardless of disease status. The expected difference in genetic markers between a case and a control should be similar to that between any two cases or between any two control subjects. In contrast, if the set is composed of susceptible SNPs, then the expected difference between a case and a control would be large, at least larger than that between any two cases or between any two controls. Here the dissimilarity is evaluated by the heterogeneity across all SNPs in this set for any two individuals, and Hamming distance is the metric considered. Notice that now the dissimilarity is evaluated between subjects’ SNP strings (the row vectors in Fig 1), not between SNP column vectors.

#### Notation and Statistics

Let *C* be the SNP-set of interest where *C* contains *K* SNPs. Let $x_{1},x_{2},\ldots ,x_{N_{1}}$ denote the genotype row vectors of the *N*_1_ subjects in the control (normal) group and $y_{1},y_{2},\ldots ,y_{N_{2}}$ denote those of the *N*_2_ subjects in the case (disease) group, where both **x**_*i*_ and **y**_*i*_ are of the form (*S*_*i*1_, *S*_*i*2_,…,*S*_*iK*_) with the same *S*_*ik*_ defined earlier. Then, the SNP genotype matrix **R**_1_ for the normal group is of dimension *N*_1_×*K*, the matrix **R**_2_ for the case group is of dimension *N*_2_×*K*, and the distance is measured between any two row vectors. Note that the stacks of **R**_1_ and **R**_2_ column-wise are the same as the stacks of **M**_1_ and **M**_2_ row-wise when the same SNP-sets are involved,

$$\left(\begin{array}{c} R_{1}\\ R_{2} \end{array}\right)=\left(\begin{array}{c} \begin{array}{c} x_{1}\\ \vdots \\ x_{N_{1}} \end{array}\\ \begin{array}{c} y_{1}\\ \vdots \\ y_{N_{2}} \end{array} \end{array}\right)=\left(\begin{array}{cccc} S_{11} & S_{12} & \cdots & S_{1K}\\ S_{21} & S_{22} & \cdots & S_{2K}\\ \vdots & \vdots & \; & \vdots \\ S_{N1} & S_{N2} & \cdots & S_{NK} \end{array}\right)=\left(S_{1},\ldots ,S_{K}\right)=\left(\begin{array}{ccc} M_{1} & : & M_{2} \end{array}\right)$$

The distance between the case and the control group is defined as the average dissimilarity between these two groups,

$$T=\frac{1}{N_{1}\times N_{2}}\sum_{\begin{array}{l} i\in \{1,\ldots ,N_{1}\},\\ j\in \{1,\ldots ,N_{2}\} \end{array}}d_{H}(h(x_{i}),h(y_{j})),$$

where *h*(**x**_*i*_) = (*h*(*S*_*i*1_), *h*(*S*_*i*2_),…*h*(*S*_*iK*_))^*t*^ = (*s*_*i*1_, *s*_*i*2_,…,*s*_*iK*_)^*t*^. The function *h* transforms the original genotype *S*_*ik*_ into a binary variable *h*(*S*_*ik*_) = *s*_*ik*_ = 1 if the subject *i* carries the minor allele at the *k*-th SNP, and 0 if not. Now the Hamming distance $d_{H}(h(x_{i}),h(y_{j}))=\sum_{k=1}^{K}I(s_{ik}\neq s_{jk})$ computes the number of SNPs for which one of the paired individuals *i* and *j* carries the minor allele while the other does not. Note that the incorporation of minor alleles in the evaluation of distance follows the rationale discussed in Wessel and Schork [23] that two individuals sharing minor alleles may reveal more similarity in their genomes than do two individuals sharing common alleles. Other functional forms can certainly be assumed for *h* if some other relationship is preferred.

The average dissimilarity within groups is

$$U=\frac{2}{N_{1}(N_{1}-1)+N_{2}(N_{2}-1)}\left[\sum_{\begin{array}{c} i,j\in \{1,\ldots ,N_{1}\}\\ i<j \end{array}}d_{H}(h(x_{i}),h(x_{j}))+\sum_{\begin{array}{c} l,m\in \{1,\ldots ,N_{2}\}\\ l<m \end{array}}d_{H}(h(y_{l}),h(y_{m}))\right].$$

Note that a *T* substantially larger than *U* implies a substantial difference in SNP-set configuration between the case and the control group. In other words, the difference *T*−*U* may provide evidence of disease association. The *p*-value for this test statistic *T*−*U* can be determined via a permutation test.

#### Clustering with Case and Control Subjects

One issue needs to be addressed when the association test adopts the SNP-sets obtained from the proposed Hamming distance-based clustering algorithm. To identify influential SNP-sets, the clustering algorithm for case-control studies should be carried out on both the case and control groups together, rather than on a single group. Taking the first four SNPs in Table 1 for example, two clusters *C*_1_ = {**S**_1_, **S**_2_} and *C*_2_ = {**S**_3_, **S**_4_} would be constructed based on 10 individuals (5 cases and 5 controls) since *d*(**S**_1_, **S**_2_) = 0 = *d*(**S**_3_, **S**_4_). This is because the Hamming distance dissimilarity algorithm groups together those SNPs which exhibit a similar pattern in both case and control groups. This includes the neutral SNPs **S**_1_ and **S**_2_ which display an identical pattern (1,1) in all 10 subjects. These two SNPs provide no information of relevance to disease association. In contrast, the other SNP-set {**S**_3_, **S**_4_} contains more information, because all cases carry (0,0) for {**S**_3_, **S**_4_} while all controls carry (1,1). The pattern in the case group alone is the same as that in the control. In other words, among these two clusters {**S**_1_, **S**_2_} and {**S**_3_, **S**_4_}, it is the latter that provides information for association. In addition, the latter set is identified only when case and control subjects are all included in the clustering algorithm. If only the control individuals are used for clustering, the cluster {**S**_1_,**S**_2_,**S**_3_,**S**_4_} would be identified with neutral SNPs included.

**Table 1:** Toy data for SNP clustering. For the purpose of illustration, the coding here for genotypes is binary.

	Cluster *C*_1_		Cluster *C*_2_	
	S_1_	S_2_	S_3_	S_4_
Case 1	1	1	0	0
Case 2	1	1	0	0
Case 3	1	1	0	0
Case 4	1	1	0	0
Case 5	1	1	0	0
Control 1	1	1	1	1
Control 2	1	1	1	1
Control 3	1	1	1	1
Control 4	1	1	1	1
Control 5	1	1	1	1

## Results

### Simulation Studies of SNP Clustering

To evaluate the proposed SNP clustering algorithm, we first simulated correlated SNPs and independent SNPs, mixed them, and then examined whether these correlated SNPs would be clustered together by the proposed algorithm. The correlated SNPs were generated with a simulation algorithm for multivariate binary variables proposed by Emrich and Piedmonte [28]. Ten correlated SNPs with minor allele frequencies (MAFs) fixed at 0.2, 0.225, 0.25, 0.275, 0.3, 0.325, 0.35, 0.375, 0.4, and 0.425 were considered, and their pairwise correlation coefficient was fixed at 0.55 or 0.3. These were then mixed with 10 or 100 other independent SNPs with MAFs ranging from 0.05 to 0.5. That is, the mixing ratios of correlated to independent variates were either 1:1 or 1:10. The genotypes of 2000 subjects were then generated based on these MAFs. The number of replications was 1000.

**Fig 2:**
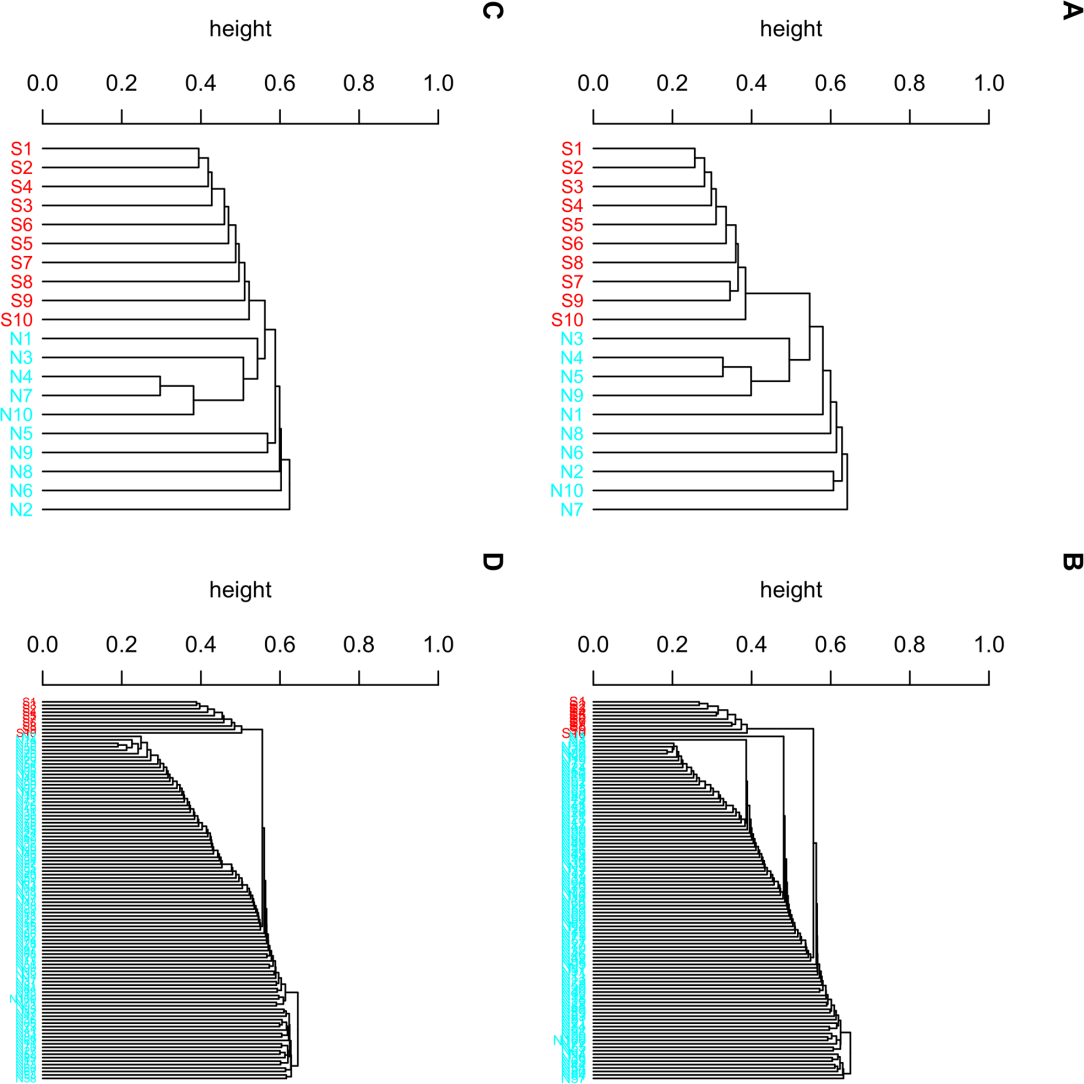
Hierarchical clustering dendrograms based on one simulated dataset. The data contained 10 correlated SNPs (S1, S2, …, S10) and 10 or 100 independent SNPs (N1, …, N10 or N1, …, N100). (A) Ten correlated SNPs with correlation of 0.55 and ten independent SNPs. (B) Ten correlated SNPs with 0.55 correlation and 100 independent SNPs. (C) Ten correlated SNPs with 0.3 correlation and ten independent SNPs. (D) Ten correlated SNPs with 0.3 correlation and 100 independent SNPs.

Under each setting, we performed the clustering algorithm and examined if the 10 correlated SNPs were grouped together. With pairwise correlation set at 0.55, the 10 correlated SNPs always formed a unique cluster under the mixing ratio of 1:1 and 1:10. When the correlation was 0.3, the percentage of this correct clustering pattern was 97.7% and 100% for the mixing ratios of 1:1 and 1:10, respectively. As for the remaining 23 (1000–977 = 23) replications, the 10 correlated SNPs were still clustered together but simultaneously mixed with an average of 3 ± 2 independent SNPs in the same cluster. Fig 2 displays the dendrograms of four typical replications.

### Simulation Studies of the SNP Association Test

To demonstrate the performance of the proposed Hamming distance-based SNP-set association test, several conditions were considered in simulation studies, including deleterious, protective, or mixed effects; and various signal-to-noise ratios. We considered a protective SNP-set containing six SNPs with odds ratios (OR) all less than 1, a deleterious SNP-set of six SNPs with OR>1, and a mixed set containing three protective and three deleterious SNPs. The values of the ORs and MAFs in the control subjects are listed in Table 2. In addition to the six causal variants (signals), other non-associated SNPs with MAFs in the range (0.05, 0.5) were also generated as neutral variants (noise). The number of neutral SNPs ranged between 6 and 90, leading to signal-to-noise ratios between 1:1 and 1:15. The numbers of cases and controls were either 100 each, or 200 each. The coding for each individual was 1 or 0 for carrying the minor allele or not. Each simulation setting contained 1000 replications with p-values derived from 1000 permutation tests.

**Table 2:** Values of ORs and MAFs in simulation studies.

Effects	OR	MAF in controls
Protection	0.83, 0.71, 0.62, 0.56, 0.48, 0.40	0.20, 0.26, 0.33, 0.37, 0.40, 0.45
Risk	1.20, 1.40, 1.60, 1.80, 2.10, 2.50	0.40, 0.36, 0.33, 0.28, 0.24, 0.20
Mixture	0.83, 0.59, 0.40, 1.20, 1.70, 2.50	0.22, 0.35, 0.43, 0.40, 0.30, 0.20

**Fig 3:**
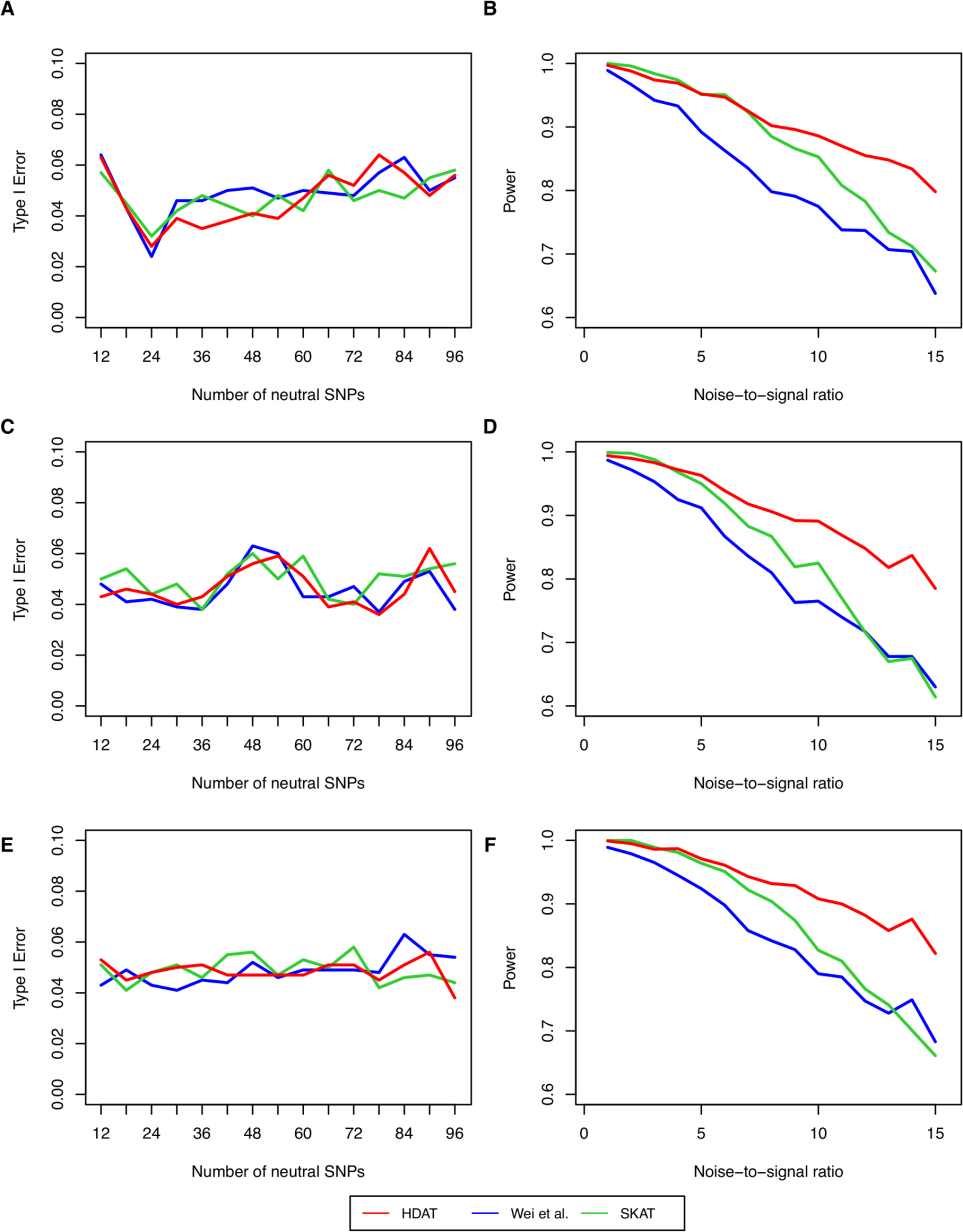
Type I error and power in simulations. Type I error (**A**, **C**, **E**) and power (**B**, **D**, **F**) of the Hamming distance-based association test HDAT (red line), the *U*-statistic (blue line) and SKAT (green line) for the SNP-set association test under different noise-to-signal ratios, and effect sizes. The X-axis stands for the numbers of neutral SNPs in (A), (C) and (E), but the noise-to-signal ratios in (B), (D) and (F). The effects of causal SNPs are deleterious in (A) and (B), protective in (C) and (D), and mixture in (E) and (F). The simulation included 100 cases and 100 controls.

Fig 3 shows the type I error and power of the Hamming distance-based association test (HDAT) for 200 subjects under different settings with the significance level 0.05. The proposed test was compared with the *U*-statistic by Wei et al. [19] and SKAT [29]. In general, all three tests had small type I errors at the nominal level. The weighted *U*-statistic had a very large type I error in comparison with the unweighted *U*-statistic, thus we considered only the latter in the following simulation comparisons. HDAT had larger power than the *U*-statistic and SKAT. The power remained above 80%, even when the signal-to-noise ratio reached 1:15, implying a consistent and reliable performance. The *U*-statistic and SKAT are more sensitive to the signal-to-noise ratio, especially when the number of individuals is not large, only 100 cases and 100 controls (Fig 3F).

### Coronary Artery Disease from WTCCC

The data, obtained from a Wellcome Trust Case Control Consortium (WTCCC) study [30], included 1926 subjects with coronary artery disease (CAD) and 2938 controls. Previous studies have shown a strong association between 9p21.3 and CAD [30–32], hence the 940 SNPs (with minor allele frequency > 0.001) on the 9p21.3 region were considered as an illustration for the Hamming distance-based clustering algorithm and association test.

**Fig 4:**
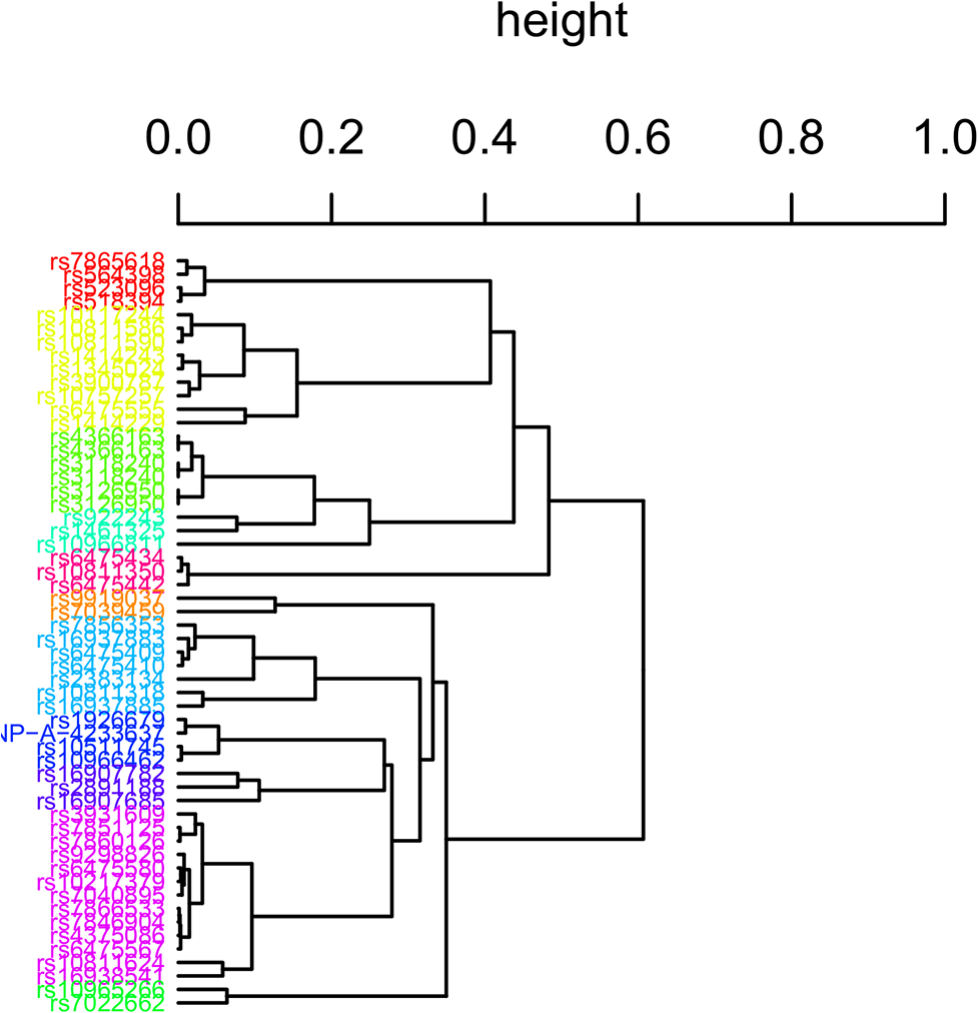
Dendrogram for the 11 selected SNP-sets. Labels indicate the SNP markers and different colors indicate different SNP-sets.

First, our proposed clustering algorithm derived 123 clusters containing at least three SNPs, and 49 clusters containing two SNPs. No cluster was composed of a single SNP. The dendrogram for all 940 SNPs is displayed in S1 Fig. The association test based on Hamming distance, HDAT, was then conducted on these clusters. The top 11 clusters with the smallest p-values (from 5000 permutations) are listed in Table 3. The dendrogram of these 11 SNP-sets is reproduced in Fig 4 with different colors. Note that most clusters contain SNPs with effects of the same direction, either protective or deleterious, as indicated in Table 3, except the sixth and the ninth set, which contain different but weak effects. After performing a Bonferroni correction for multiple tests, the first set with four deleterious SNPs remains significant (p<0.0005 in 5000 permutations). Alternatively, selecting the top 5, or top 10%, relative heights *D*(**S**_*k*_) to determine the clusters for the subsequent association test results in the same SNP-set being significant.

**Table 3:** The compositions and size of the top 11 SNP-sets with the smallest *p*-values. SNPs in boldface indicate protective effect from single-marker test (OR<1) and the rest indicate deleterious effect (OR>1).

SNP-set	List of SNPs	Number of SNPs	*p*-value^a^
1	**rs7865618, rs523096, rs564398, rs518394**	4	0.0002^b^
2	rs9919037, rs7039459	2	0.0010
3	**rs10117244, rs10811586, rs6475555, rs1414243, rs3900787, rs10811590, rs1345024, rs1414229, rs10757257**	9	0.0040
4	**rs4366163, rs3118240, rs3126950**	3	0.0064
5	rs10811318, rs16937885	2	0.0078
6	**rs4366163, rs922243, rs3118240,** rs10966811, **rs1461325, rs3126950**	6	0.0186
7	rs7856353, rs2383134, rs16937883, rs6475409, rs6475410, rs10811318, rs16937885	7	0.0596
8	rs1926679, rs10511745, rs10966462, SNP-A-4233637^c^	4	0.0730
9	rs16907782, rs2891188, **rs16907685**	3	0.0770
10	rs3931609, rs10811624, rs7851125, rs9298826, rs6475580, rs7040895, rs7860126, rs7866533, rs7846904, rs10217379, rs16938541, rs4375086, rs6475567	13	0.0866
11	**rs6475434, rs10811350, rs6475442**	3	0.0902

^a^: *p*-values were obtained from 5000 permutations.

^b^: This set remains statistically significant after Bonferroni correction.

^c^: No rs number was found for this SNP and thus it was labeled with an Affymetrix ID.

This significant cluster contains 4 SNPs, rs7865618, rs523096, rs564398, and rs518394, all in the *MTAP* gene, which has been reported to be involved in homocysteine metabolism [30–32]. This SNP-set spans 11.9kb and includes only protective SNPs. Their minor alleles all carry significant protective effects (p-values for single marker tests all < 1e-5). In addition, these variants have been investigated in the laboratory and found to be significantly associated with disease in other GWAS [31].

For the 172 (= 123+49) clusters identified by the Hamming distance based clustering algorithm, we also applied Wei et al.’s [19], SKAT [28], and HDAT with 1000 permutations. Only three sets reached significance with all three association tests. These three sets were the top three SNP-sets identified by HDAT, also listed in the top of Table 3. Because Wei et al.’s method is time consuming, here we only performed 1000 permutations in the test of each SNP-set. This number is not large enough to carry out a Bonferroni correction for this CAD study. The purpose is simply to demonstrate the similar findings reached under the three tests.

We also applied the k-mode [15] and Zhang et al.’s [16] method to cluster these 940 SNPs. It took 5.88 hours for the k-mode to perform clustering, but only 26.5 minutes for our Hamming distance clustering algorithm to complete the computation, while Zhang’s method failed to converge because the data size was too large. The first row in Table 4 lists the respective computational times.

**Table 4:** Numbers are the run time (average and standard deviation of 10 repetitions) under the Hamming distance-based clustering algorithm (HD), k-mode, and Zhang’s method for the three applications. For the first three applications, the run time was assessed with single-threaded computation; while the time for the last application was under parallel computation and single-threaded computation in R.

Application	HD	k-mode	Zhang
CAD in WTCCC(940 SNPs, 4864 subjects)	26.5±0.88 min.	539±122 min.	—
ENCODE(7538 SNPs, 269 subjects)	43±0.6 sec.	1400±376 sec.	—
Soybeans(35 attributes, 47 beans)	0.11±0.01 sec.	0.77±0.16 sec.	2.34±0.05 sec.
CAD in WTCCC(4000 SNPs, 4864 subjects)	18.27 hours (parallel)	—	—
	52.44 hours (single-threaded)	—	—

#### HDAT on LD Blocks

Alternatively, with Haploview, we constructed 145 LD blocks containing at least two SNPs based on the same 940 SNPs. These blocks were determined based on the correlation among neighboring loci; where SNPs of deleterious and protective effects can both appear in the same block. All 145 blocks were tested with HDAT. S1 Table lists ten blocks with smaller p-values. These blocks were significant with p<0.05 but none reached significance after Bonferroni correction. Two SNPs (rs523096, rs518394) in the first LD block also appeared in the first SNP-set of the Hamming distance clusters in Table 3.

#### Hamming Distance Clusters and LD Blocks

For reference, we displayed in S2 Fig the linkage disequilibrium measure *r* ^2^ for the Hamming distance clusters. The 4 SNPs in our first Hamming distance cluster, though highly correlated with each other (*D*′>0.96, *r* ^2^>0.87), were separated into two different LD blocks, due to another SNP (rs10758264) locating between them and its weak LD with these 4 SNPs. Another example for the difference between LD blocks and Hamming distance clusters is the SNP-set 6 in Hamming distance clusters. Inside this cluster, SNP rs10966811 is not in close linkage with other SNPs (*r* ^2^ in 0.21~0.46, *D*′ in 0.51~0.83), and thus not contained in the LD block 2 (The LD block numbered 2 in S1 Table). Only 4 close SNPs (rs1461325, rs3126950, rs4366163, rs3118240) from this Hamming distance cluster are included in the LD block 2. In addition, the spans of these clustered SNP-sets can vary greatly. For instance, the SNP-set 11 ranges 3.4 kilobase pairs; while the SNP-set 6 ranges 183.6 kb. These sets may serve as a complementary option or reference if one decides to combine or prune LD blocks.

In contrast to the test with all SNP-sets, we applied the gap statistic to determine the number *K* of clusters under every clustering algorithm discussed above. However, based on the gap statistic, no best value for *K* was selected in the range of 1 to 250. Again, such large data size induces extensive computation and hence cannot be accommodated by most traditional methods.

### Simulation Studies for Combined Procedures

The statistical significance of any association test on a large SNP-set can only imply a possible relationship between this whole set and the disease of interest. A finer range or locations of the susceptible genes may still remain unknown. With the Hamming distance clustering algorithm, a larger set can be divided into several smaller SNP-sets whose significance can provide more information as where these susceptible genetic markers may locate. In this simulation, we investigated if the performance of any of the association tests (HDAT, *U* statistic, and SKAT) can be improved by testing on the Hamming distance clusters.

The same procedures for generating the genotypes in the simulation studies for SNP association tests were adopted. The numbers of cases and controls were 100 each, and the number of replications was 1000. In each replication, the beginning SNP-set containing 6 signal (risk) SNPs and 90 neutral SNPs was constructed and tested with every one of the three tests. The powers were 77%, 63%, and 67%, respectively. We refer to these as the overall powers.

**Fig 5:**
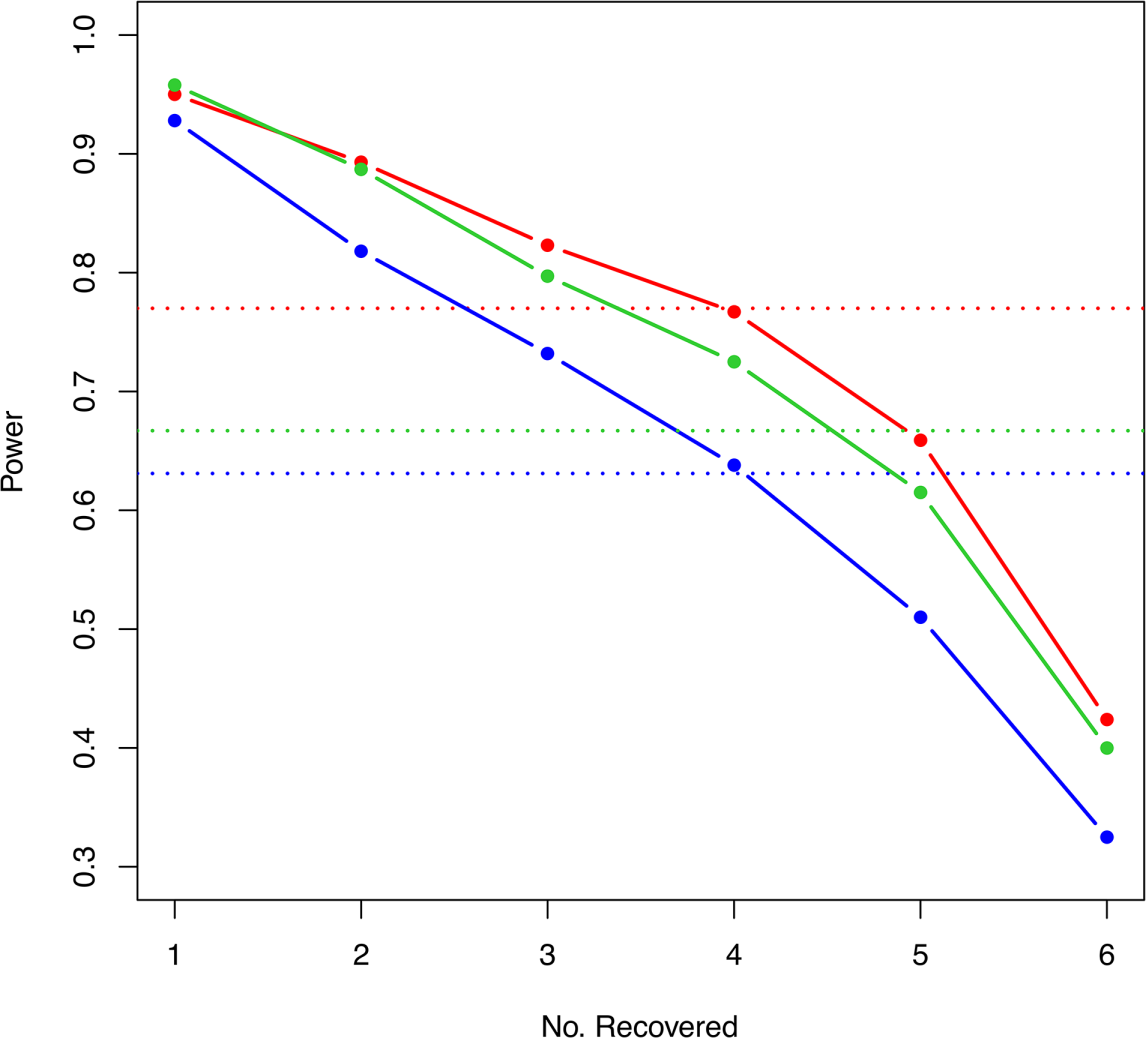
Power calculation for combining Hamming distance clustering algorithm and association test. Solid lines are for clustering+HDAT (red), clustering+*U*-statistic (blue) and clustering+SKAT (green); while dotted lines are for the tests on the original complete set (termed as the overall power).

For the purpose of comparison, each one of the large sets underwent the Hamming distance clustering algorithm first, followed by each of the three tests on every one of the resulting clustered SNP-sets. We then examined if any clustered SNP-sets containing signal SNPs were successfully detected with statistical significance, as well as the number of signal SNPs detected in each replication. The solid lines in Fig 5 show that, for any of the three association tests, carrying out the test on Hamming distance clustered sets can reach a power larger than 90% (95% for HDAT, 93% for *U* statistic, and 96% for SKAT) in identifying at least one susceptible SNP marker, and larger than 70% (82% for HDAT, 73% for *U* statistic, and 80% for SKAT) in identifying at least three markers. If the overall power was taken as the baseline (dotted line in Fig 5), then testing on the clustered sets can provide more information on genomic range of at least 4 SNPs for each one the three tests.

Note also that, combining the clustering and HDAT (red solid line) has a slightly better performance than combining the clustering and SKAT (green solid line). The Hamming clustering algorithm improved SKAT more (77% vs. 0.82% for HDAT and 67% vs. 80% for SKAT), because SKAT is sensitive to the ratio of noise to signal SNPs (shown in earlier section and Fig 3). The type I errors for the three combination tests were all reasonable (5.2%, 5.5%, and 5.0%, respectively).

This experiment implies, in addition to the better performance of HDAT over the other two tests, that the Hamming distance clustering algorithm can help to identify smaller SNP-sets for association tests like *U* statistic and SKAT, and thus improves the ability to confine or localize genetic regions for future studies.

## Discussion

The proposed SNP clustering algorithm based on the Hamming distance dissimilarity measure not only works faster than current existing methods, it is also free from the constraint that SNP-sets are formed by neighboring SNPs. This algorithm can also be used to determine whether several given SNP-sets should be clustered together. Additionally, this clustering algorithm can separate protective from deleterious genetic variants, resulting in SNP-sets containing components with effects of the same direction. The ability to produce such sets would be useful as some association tests work only for SNPs with effects of the same direction. When illustrating this algorithm, we started with original SNP genotypes to gradually form SNP-sets of various sizes. In other words, this proposed procedure can be employed directly on SNP-sets that are pre-determined based on certain functional characteristics. For instance, if different known relationship, such as inhibition or activation, between members is to be specified in this clustering procedures, then the coding should be modified accordingly. One example would be to replace {0, 1, 2} with {1, 2, 3} for tumor suppressor genes (or protective loci) and {-1, -2, -3} for oncogenic markers.

Other applications of this algorithm are the construction of population structures and clustering of multi-categorical variables.

### Construction of Population Structures

**Fig 6:**
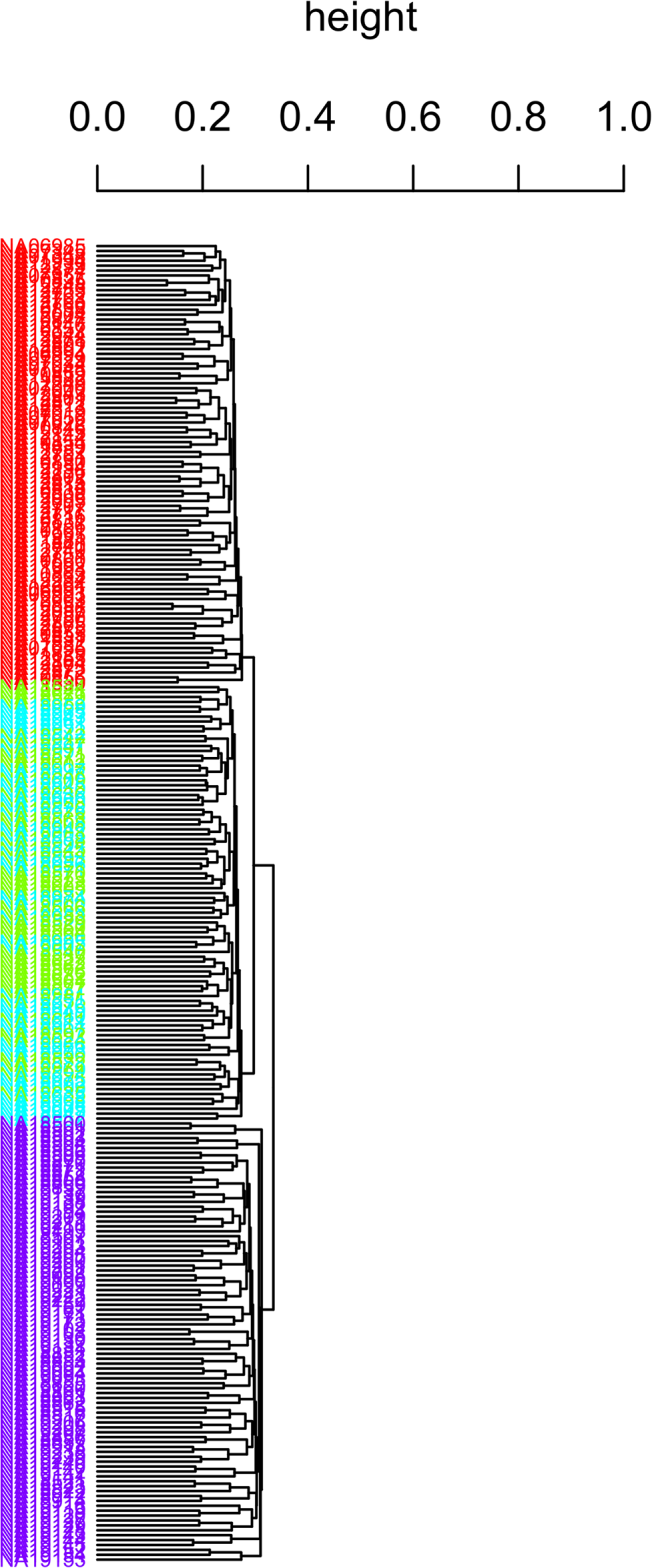
Dendrogram for the HapMap ENCODE database. Different colors represent different populations, red for CEU, blue for Japanese, green for Chinese, and purple for Yorba.

**Fig 7:**
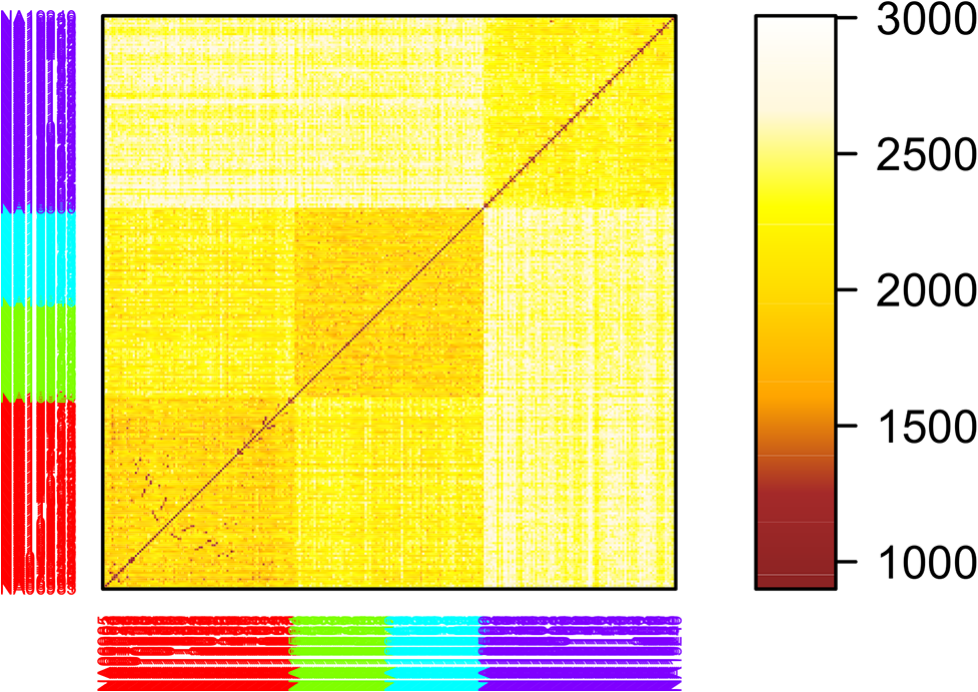
Heatmap of the corresponding Hamming dis-similarity matrix for the HapMap ENCODE database. Different colors represent different populations, red for CEU, blue for Japanese, green for Chinese, and purple for Yorba.

The Hamming distance-based clustering algorithm can be used as a tool to elucidate the underlying population structure from genomic SNP data. We illustrate this property with data from HapMap ENCODE database [33–34] containing a total of 7538 SNP genotypes from 269 subjects (90 Yorba, 44 Japanese, 45 Han Chinese, and 90 CEU Utah residents with northern and western European ancestry). The clustering procedure was applied on the 7538×269 SNP matrix, and the column variables (individuals) were clustered. Fig 6 displays the results where different colors represent different populations. Three clusters were identified, one for CEU, one for Japanese and Chinese together, and one for Yorba. These three clusters are fairly distinct from each other. However, these SNPs cannot clearly differentiate Japanese from Chinese. The heatmap of the corresponding Hamming dis-similarity matrix is displayed in Fig 7. The pattern between groups does differ from the pattern within any group (squares in diagonal direction in Fig 7). The proposed clustering algorithm took 43 seconds to construct three clusters; while the k-mode took 23 minutes. For Zhang’s method, the size was again too large to compute (computation times in the second row of Table 4).

### Accommodation of Multi-categorical Variables

**Fig 8:**
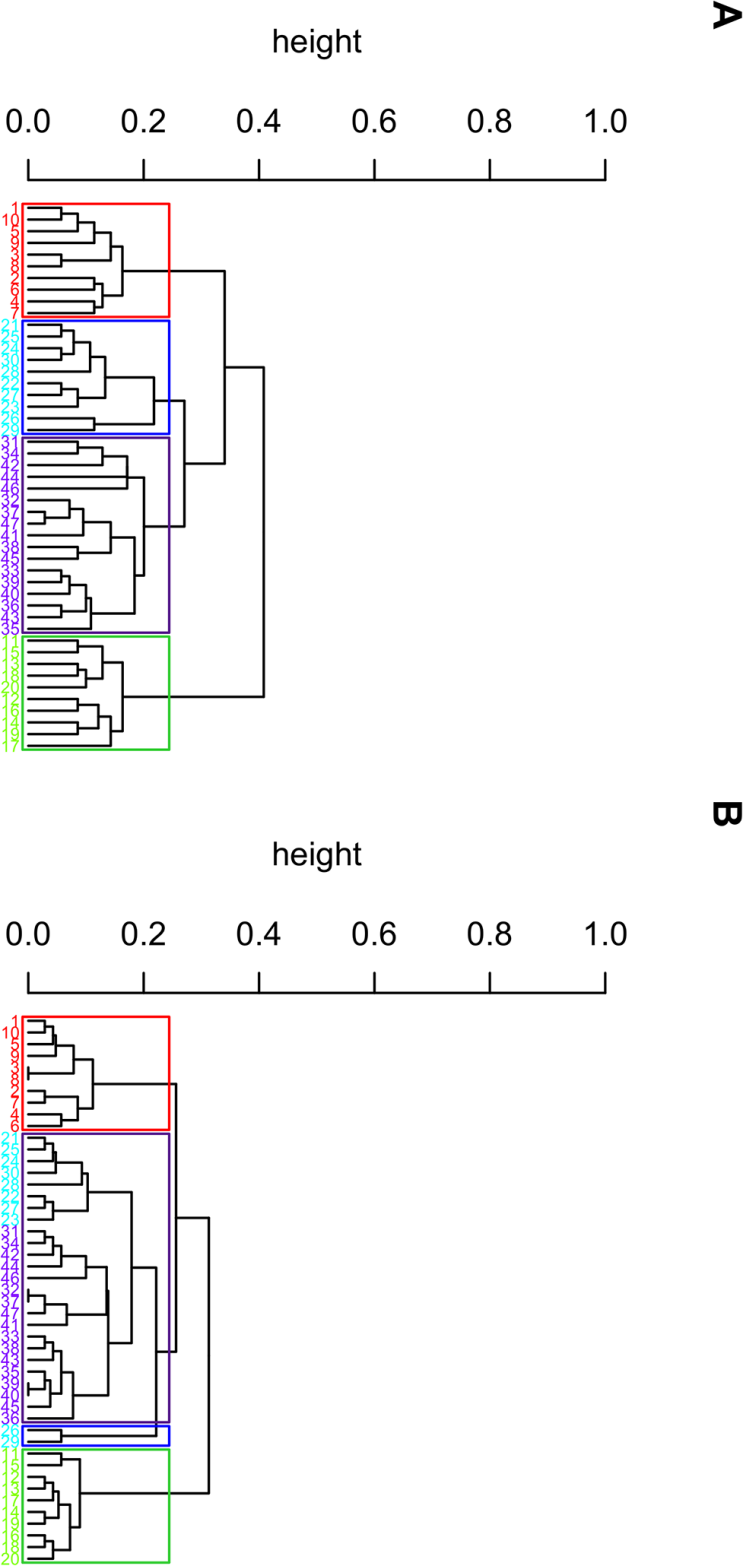
Dendrogram for the soybean data. The four colors are for the 4 true classes. (A) The original categories are used as the coding for each variable. (B) The binary coding is considered for each variable.

In previous sections, when it is of interest to cluster SNPs or SNP-sets, we used the same coding system (1/0 coding indicating with and without minor alleles, or 0/1/2 for the number of minor alleles) for all SNPs. However, if this clustering algorithm is used to cluster variables of different levels, then the Hamming distance-based algorithm can be extended easily. In other words, the algorithm can handle variables with different numbers of categories. The following soybean data include 47 individual soybeans, each being 1 of 4 classes, and 35 variables (attributes) related to the appearance, growing environment and date of bloom of each bean, with the number of levels for each variable ranging from two to seven [35]. Using the original levels for each variable, the clustering algorithm successfully identified the four actual classes of clusters (Fig 8A). In contrast, two beans would be mis-clustered when the binary coding (0 for the first level and 1 for the rest) was used (Fig 8B).

### Computational Burden

In previous sections we have proposed a clustering algorithm and an association test, both based on Hamming distance measure. The clustering algorithm focuses on the similarity between SNP vectors (the column vector **S**_*k*_), where each vector contains the genotypes of a specific SNP for all subjects. The test, in contrast, examines the similarity between subject vectors (the row vector), where each vector denotes the genotypes of all SNPs for a specific individual. The computational burden, therefore, depends on the number of variables to be compared for similarity; it is the number of SNPs in the case of the clustering algorithm and the number of subjects in the case of the test. The order of the computational complexity is discussed in S2 Text. Here we examined first the run time for Hamming distance clustering algorithm. In Table 4, it is clear that clustering 940 SNPs took longer (26.5 minutes) than clustering the ENCODE 269 subjects (43 seconds). If the number of variables to be clustered increases to 4000, such as the case of clustering 4000 SNPs from 4864 subjects (the last row in Table 4), then the computation takes 52.45 hours for single-threaded computation in an ordinary desktop computer with Intel Core i7-4770 processor (3.40 GHz) and 32 GB RAM. With such volume of data, we then carried out 7-CPU parallel computation with packages *doSNOW* and *foreach* in R, leading to 18.27 hours (Table 4). Next, we investigated how sensitive the run time for HDAT is as we change the number of subjects to be compared and the number of permutations in the test. For illustration, we selected randomly 1000 subjects from the WTCCC study, along with their first 5000 SNP genotypes in 9p or all SNPs on chromosome 9, and next performed HDAT with either 1000 or 5000 permutations with parallel computation. Table 5 lists the corresponding run time under each scenario. When all 19948 SNPs on chromosome 9 were included, it took 121.56 seconds for 1000 subjects and 72 minutes for 4864 subjects to complete the test, with the number of permutations set at 1000. The increase in the number of subjects has a greater influence on the computational burden. When handling data of a larger scale like GWAS, either performing parallel computations, adopting C-alike programming languages, or considering graphics processing unit (GPU) computing will surely enhance substantially the computational performance.

**Table 5:** Numbers are the run time (in seconds, s, or minutes, m) of HDAT, using parallel computation in R, under different numbers of subjects, SNPs, and permutations.

		Run time with 1000 or 5000 permutations	
No. of subjects	No. of SNPs	1000	5000
1000	5000	33 sec.	76 sec.
1000	19948	122 sec.	163 sec.
4864	1000	504 sec.	2473 sec.
4864	5000	19 min.	44 min.
4864	19948	72 min.	107 min.

### Extension to Rare Variants

**Fig 9:**
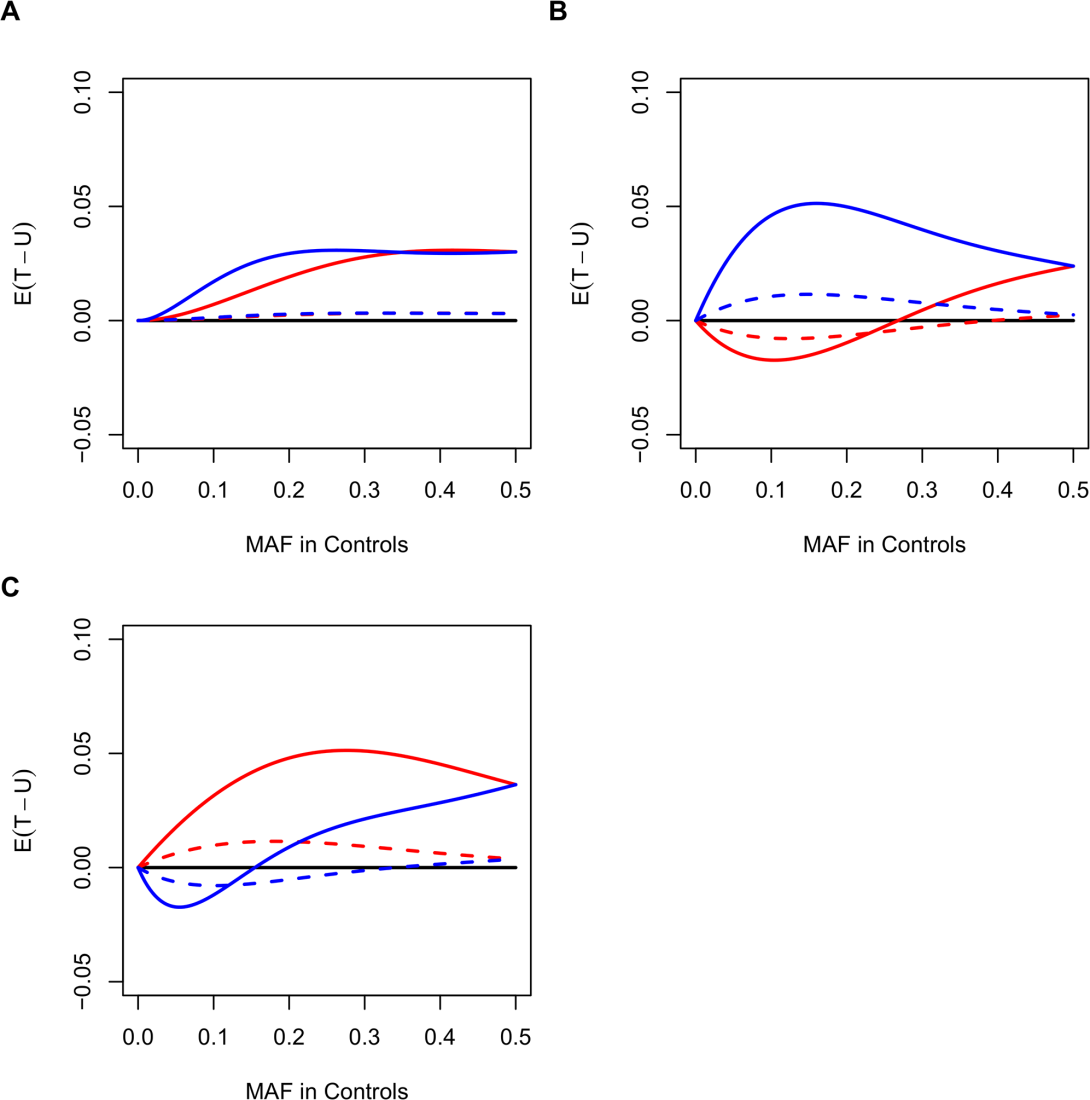
Expected values of the test statistic. Expected values of the test statistic under different MAFs in controls, ORs, and case-to-control ratios based on one single SNP. (A) Case-to-control ratio is 1:1 (1000:1000). (B) 1:1.5 (1000:1500). (C) 1.5:1 (1500:1000). Red solid line for protective SNPs with OR = 0.5, red dashed line for OR = 0.8, blue solid line for OR = 2, and blue dashed line for OR = 1.25.

In addition to the common causal variants, this test may be extended to rare causal variants. When dealing with rare variants, the small frequency of individuals carrying the minor allele greatly impacts the difference between the two statistics *T* and *U*. Taking a deleterious causal rare variant for example, since it is rare only a small proportion of individuals in the case group will carry this variant, making the value of the Hamming distance measure *U*_*cs*_ in the case group similar to *T*. To increase the statistical power in this case, inclusion of a larger group of controls would correspond to a within-group Hamming distance *U*_*cn*_ that would balance *U*_*cs*_, making *U* (the weighted combination of *U*_*cs*_ and *U*_*cn*_) move away from *T*. Fortunately, when the allele represents risk, it becomes easier to find more controls than cases. Such recruitment would not be hard. The influence of the increase in control samples can be observed in the difference in magnitude between the blue lines in Fig 9A and 9B. Similarly, when the causal rare variant is protective, then only a small proportion of subjects in the control group will carry this variant, leading *U*_*cn*_ and *T* to share a similar magnitude. Therefore, following the same argument above, a larger sample size in the case group will enlarge the difference between *U* and *T*. This effect on *T*−*U* when relatively more cases are involved in the study can be seen by comparing the red lines in Fig 9A and 9C. Our team is currently working on this topic with modifications on variant-specific weights.

## Conclusions

As discussed in previous sections, our clustering algorithm saved more computational time than existing methods. For large data size, our algorithm easily outperforms the others (first two rows in Table 4). Even for data of smaller size like the soybean data, our algorithm took only 0.1 second, while k-mode took 1 second and Zhang’s took 2 seconds (the third row in Table 4). Another advantage of our proposed clustering algorithm is that applying the Hamming distance-based algorithm with both control and case observations before conducting an association test can produce SNP-sets containing SNPs with effects of the same direction. The homogeneity of SNP effects may warrant further biological validation studies, and will ensure statistical power in certain association analyses [36].

Our second proposal, the association test HDAT, can accommodate a large number of SNPs in one test, while allowing for SNPs in the same set to share the same effect or to have mixed effects. If this test is to be used on a pre-defined SNP-set in which the effects may be mixed, this association test can outperform other tests, especially when the majority of SNPs are neutral. When sample size reaches 400, all three tests perform equally well (S3 Fig), regardless of the noise-to-signal ratio. This HDAT can be used on other previously defined SNP sets. For instance, this test can be applied on haplotype blocks that have been constructed based on *D*′ or *r* ^2^ from linkage disequilibrium analysis. In other words, the test can be employed along with other clustering algorithms. The R code for clustering SNP-sets and testing for disease association is available at http://homepage.ntu.edu.tw/~ckhsiao/HammingDistance/HD.htm.

This research was concerned mainly with common variants. Certain issues remain to be resolved. First, when rare variants are of major interest, we have recommended to include different numbers of cases and controls for the association test. Much of the detailed allocations and adjustment for minor allele frequencies are currently under study. Second, if admixture populations exist in association studies, it is not known whether one should cluster the subjects before or after the association test. Intuitively, handling the population stratification first before conducting HDAT on the case and control groups of the same population should be recommended. However, the stratified populations to be tested may be of smaller sizes, resulting in unstable findings. A balance between the problem of population stratifications and the choice of sample size for statistical power should be evaluated. Third, the association test considered in this article only deals with genetic markers. No covariates have been discussed. If the covariates are available and can be transformed into categorical variables, then they can be considered as pseudo markers and their similarity can be measured with Hamming distance. Such treatment, however, may not work on continuous explanatory variables. Our next research will extend the Hamming distance measure to covariates, so that effects of the genetic markers as well as other environmental factors can be examined via regression models.

## Supporting Information

S1 Fig The dendrogram for CAD data from WTCCC. Different colors represent the 11 selected SNP-sets with smaller p-values under HDAT.
(TIFF)

S2 Fig The linkage disequilibrium measure *r* ^2^ for the Hamming distance clusters. (TIFF)

S3 Fig Type I error and power in simulations with 400 subjects. Type I error in (A), (C), (E) and power in (B), (D), (F) of the Hamming distance-based association test HDAT (red line), the *U*-statistic (blue line) and SKAT (green line for the SNP-set association test under different noise-to-signal ratios, and effect sizes. The X-axis stands for the numbers of neutral SNPs in (A), (C), and (E), but the noise-to-signal ratios in (B), (D), and (F). The effects of causal SNPs are deleterious in (A) and (B), protective in (C) and (D), and mixed in (E) and (F). This simulation included 200 cases and 200 controls.
(TIFF)

S1 Table The association test HDAT on LD blocks. (DOCX)

S1 Text A hypothetical dendrogram and computation of *H*_*k*(*i*)_ and *D*(S_*k*_). (DOCX)

S2 Text Computational complexity for the Hamming distance clustering algorithm and association test. (DOCX)
