# Supplementary figures and images for: Using Hamming Distance as Information for SNP-Sets Clustering and Testing in Disease Association Studies
Source: PLoS One. 2015 Aug 24;10(8):e0135918. doi: 10.1371/journal.pone.0135918 (PMC4547758; doi:10.1371/journal.pone.0135918)

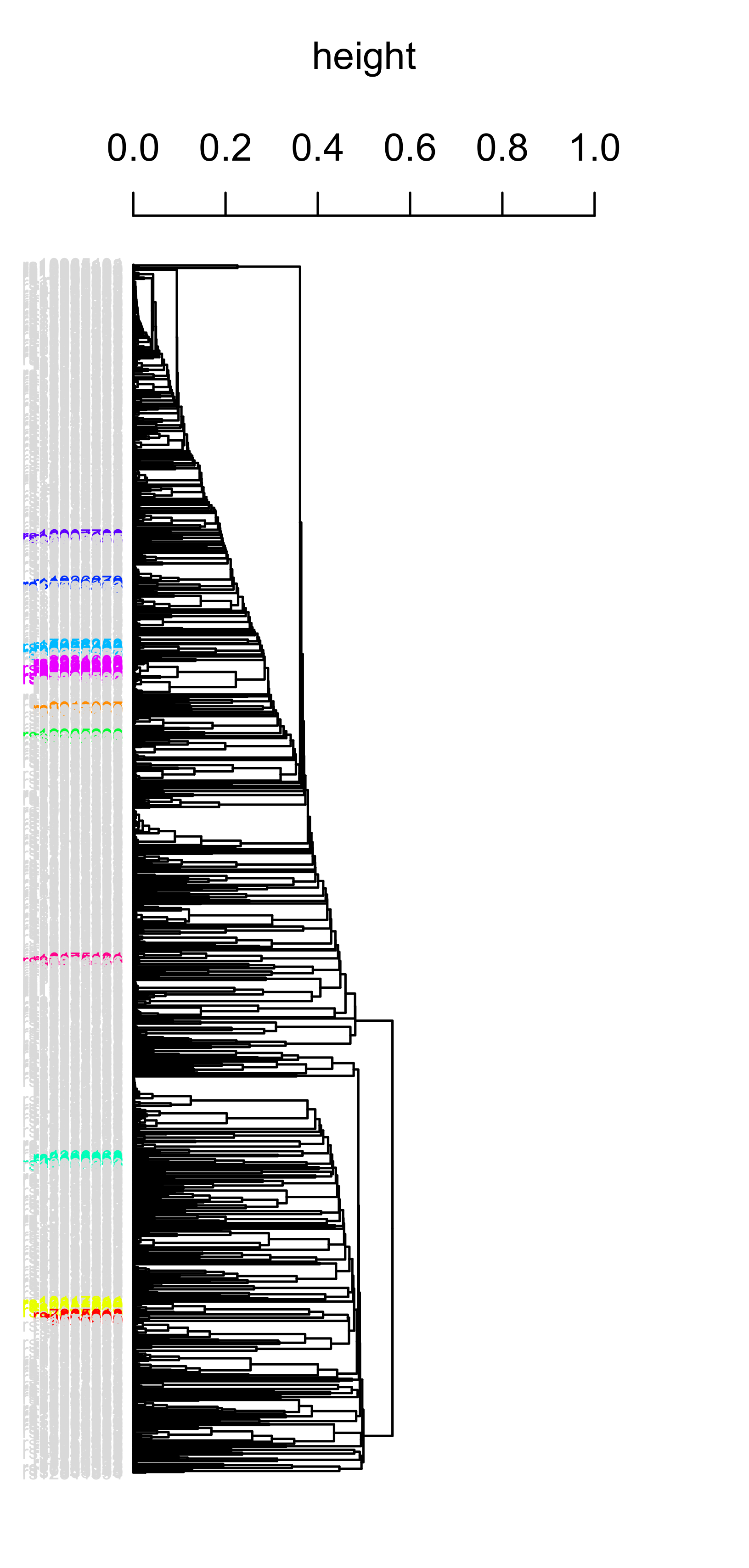

Supplement: S1 Fig — Different colors represent the 11 selected SNP-sets with smaller p-values under HDAT. (TIFF) [file pone.0135918.s001.tiff]

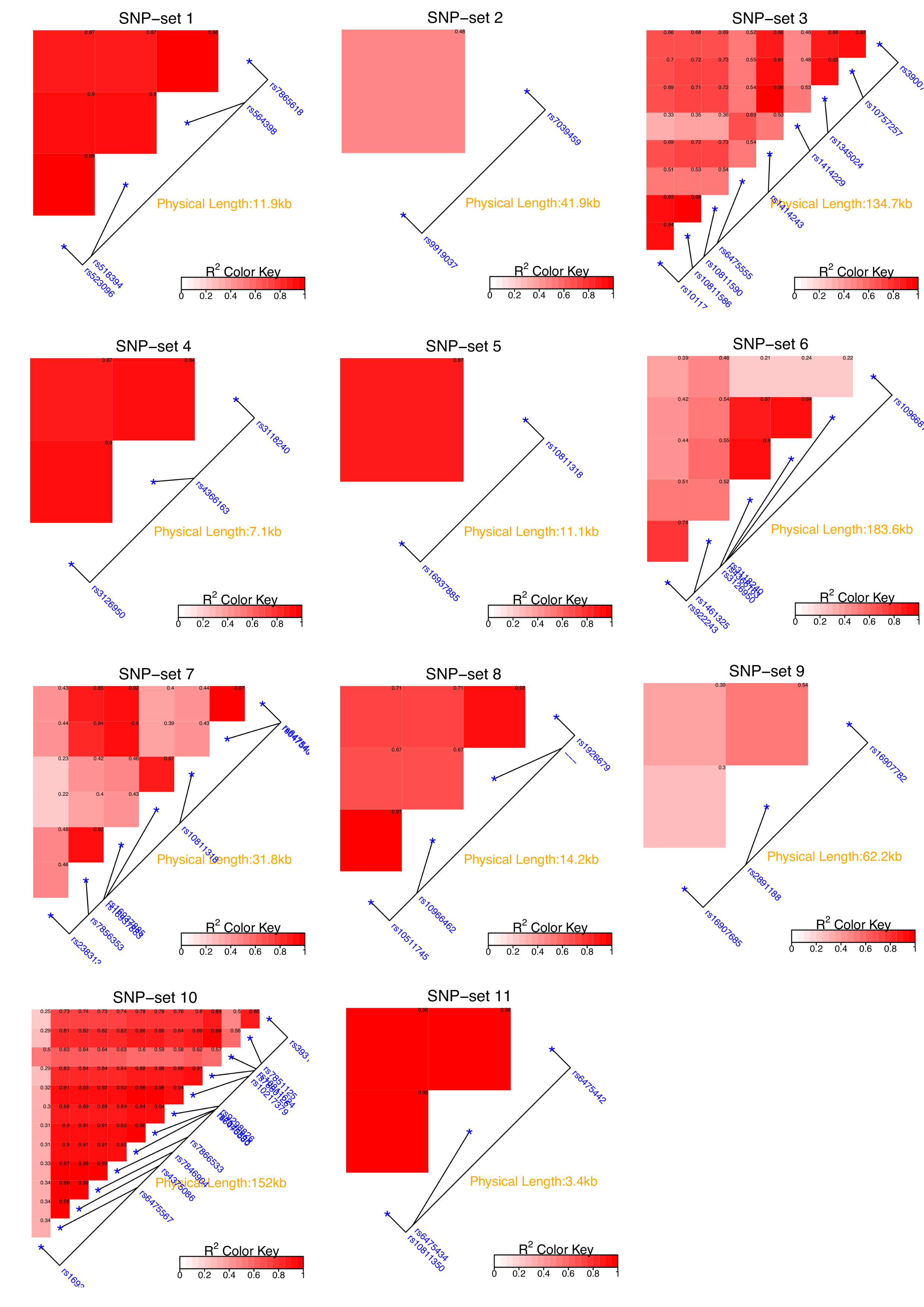

Supplement: S2 Fig — (TIFF) [file pone.0135918.s002.tiff]

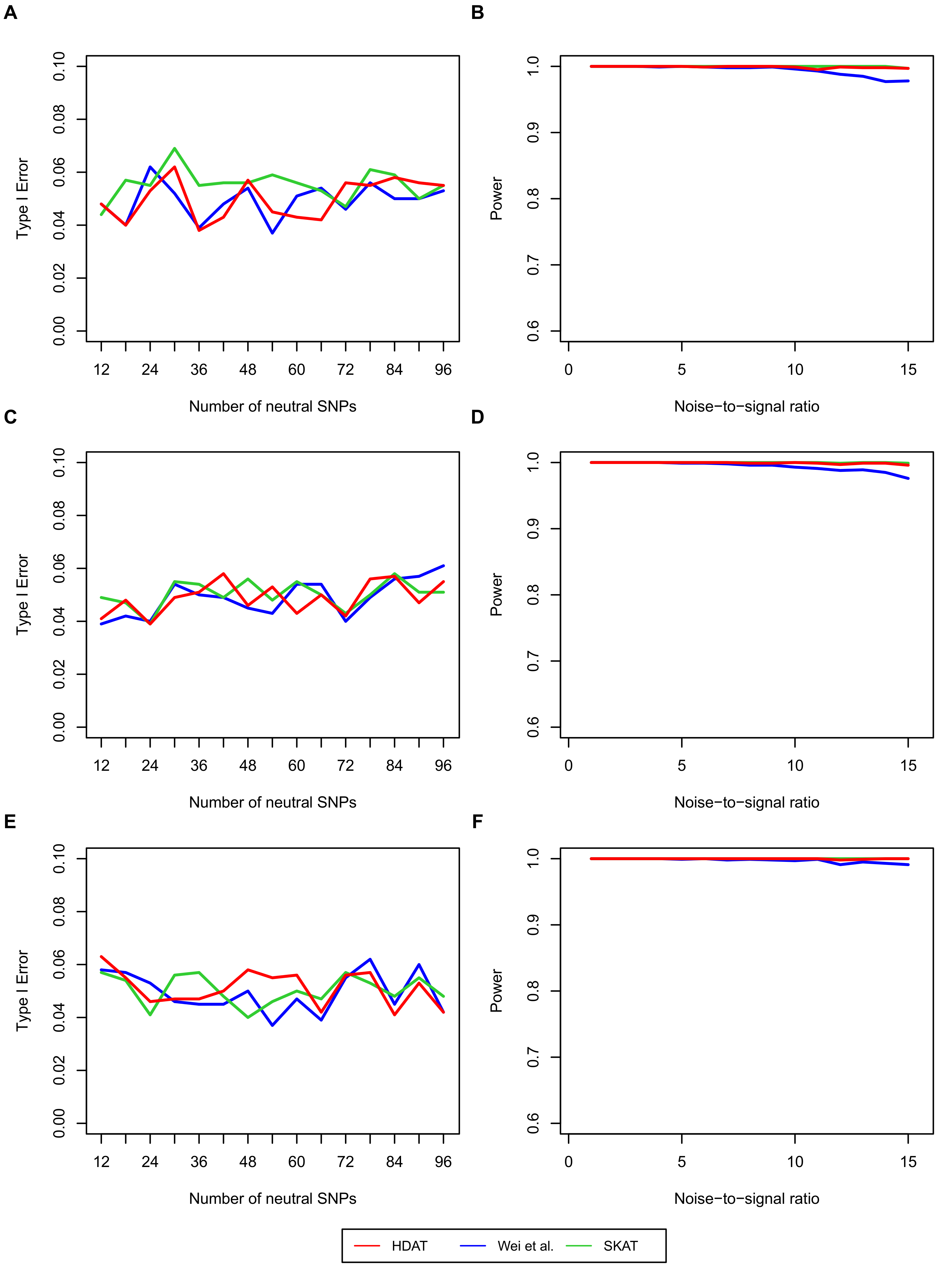

Supplement: S3 Fig — Type I error in (A), (C), (E) and power in (B), (D), (F) of the Hamming distance-based association test HDAT (red line), the U-statistic (blue line) and SKAT (green line for the SNP-set association test under different noise-to-signal ratios, and effect sizes. The X-axis stands for the numbers of neutral SNPs in (A), (C), and (E), but the noise-to-signal ratios in (B), (D), and (F). The effects of causal SNPs are deleterious in (A) and (B), protective in (C) and (D), and mixed in (E) and (F). This simulation included 200 cases and 200 controls. (TIFF) [file pone.0135918.s003.tiff]
