# Supplementary material for: Using Hamming Distance as Information for SNP-Sets Clustering and Testing in Disease Association Studies
Source: PLoS One. 2015 Aug 24;10(8):e0135918. doi: 10.1371/journal.pone.0135918 (PMC4547758; doi:10.1371/journal.pone.0135918)
Supplement: S1 Table — (DOCX) [file pone.0135918.s004.docx]

**Table S1**: The compositions and size of the top 10 SNP-sets with the smallest *p*-values. SNPs in red indicates protective effect from single-marker test (OR<1) and blue for deleterious effect (OR>1). None remains statistical significant after Bonferroni correction.

| LD block | List of SNPs | Number of SNPs | *p*-value |
| --- | --- | --- | --- |
| 1 | rs523096^a^, rs518394^a^ | 2 | 0.0016 |
| 2 | rs2783960, rs1461325^b^, rs3126950^b^,  rs4366163^b^, rs3118240^b^ | 5 | 0.0050 |
| 3 | rs7865524, rs10811474 | 2 | 0.0134 |
| 4 | rs16937883^c^, rs16937885^c,d^, rs10811318^c,d^, rs6475409^c^, rs6475410^c^, rs10964439, rs1333783 | 7 | 0.0180 |
| 5 | rs10465048, rs4977395, rs7039459^d^ | 3 | 0.0244 |
| 6 | rs6475580, rs7040895, rs9298826, rs10757257, rs10217379, rs7860126, rs10811624, rs7851125, rs7027989, rs4345650, rs3900787, rs3931609, rs7851133 | 13 | 0.0368 |
| 7 | rs2891188, rs2026037 | 2 | 0.0402 |
| 8 | rs507004, rs1375291 | 2 | 0.0504 |
| 9 | rs12685552, rs16907782 | 2 | 0.0586 |
| 10 | rs1319332, rs9919037^e^, rs403468 | 3 | 0.0640 |

^a^: These SNPs also appeared in the 1st set of Hamming distance clusters in Table 3.

^b^: Theses SNPs also appeared in the 6th set of Hamming distance clusters in Table 3.

^c^: Theses SNPs also appeared in the 7th set of Hamming distance clusters in Table 3.

^d^: Theses SNPs also appeared in the 5th set of Hamming distance clusters in Table 3.

^e^: Theses SNPs also appeared in the 2nd set of Hamming distance clusters in Table 3.
