# Supplementary material for: Using Hamming Distance as Information for SNP-Sets Clustering and Testing in Disease Association Studies
Source: PLoS One. 2015 Aug 24;10(8):e0135918. doi: 10.1371/journal.pone.0135918 (PMC4547758; doi:10.1371/journal.pone.0135918)
Supplement: S1 Text — (DOCX) [file pone.0135918.s005.docx]

**Text S1** A hypothetical dendrogram and computation of $H_{k(i)}$ and $D(\mathbf{S}_{k})$

For the SNP $\mathbf{S}_{k}$, it is included in clusters $C_{k(1)}\subset C_{k(2)}\subset C_{k(3)}\subset C_{k(4)}$ through nodes A, B, and C in the following dendrogram, where

$$\begin{matrix} C_{k(1)}=\{\mathbf{S}_{k}\}, \\ C_{k(2)}=\{\mathbf{S}_{k},\mathbf{S}_{2}\}, \\ C_{k(3)}=\{\mathbf{S}_{k},\mathbf{S}_{2},\mathbf{S}_{3}\}, \\ C_{k(4)}=\{\mathbf{S}_{k},\mathbf{S}_{2},\mathbf{S}_{3},\mathbf{S}_{4},\mathbf{S}_{5}\} \end{matrix}$$

The height of node A (vertical distance from bottom to node A) is the Hamming distance between $\mathbf{S}_{k}$ and $\mathbf{S}_{2}$,

$$\begin{matrix} d_{H}(\mathbf{S}_{k},\mathbf{S}_{2})=d(\{\mathbf{S}_{k}\},\{\mathbf{S}_{2}\}) \\ \text{ }=d(C_{k(1)},C_{k(2)}-C_{k(1)}) \\ \text{ }=H_{k(1)} \end{matrix}$$

The height of the node can be considered as the “height” of the new cluster $C_{k(2)}$ as well. Similarly, the height of node B is

$d(\{\mathbf{S}_{k},\mathbf{S}_{2}\},\{\mathbf{S}_{3}\})=d(C_{k(2)},C_{k(3)}-C_{k(2)})=H_{k(2)}$.

The height of node C is $H_{k(3)}=d(C_{k(3)},C_{k(4)}-C_{k(3)})=d(\{\mathbf{S}_{k},\mathbf{S}_{2},\mathbf{S}_{3}\},\{\mathbf{S}_{4},\mathbf{S}_{5}\})$.

Hence, the maximum “relative height” is defined as

$D(\mathbf{S}_{k})=\max\{H_{k(2)}-H_{k(1)},H_{k(3)}-H_{k(2)}\}=H_{k(3)}-H_{k(2)}$.

That is, the cluster $C_{k(3)}$ is selected for SNP $\mathbf{S}_{k}$. Similar calculation will select the same cluster $C_{k(3)}$ for SNPs $\mathbf{S}_{2}$ and $\mathbf{S}_{3}$ as well.
