# Supplementary material for: Using Hamming Distance as Information for SNP-Sets Clustering and Testing in Disease Association Studies
Source: PLoS One. 2015 Aug 24;10(8):e0135918. doi: 10.1371/journal.pone.0135918 (PMC4547758; doi:10.1371/journal.pone.0135918)
Supplement: S2 Text — (DOCX) [file pone.0135918.s006.docx]

# Computational Complexity

## Clustering Algorithm

For a genomic region containing $K$ SNPs $\{\mathbf{S}_{1},\mathbf{S}_{2},...,\mathbf{S}_{K}\}$, Hamming distance dissimilarity between all pairs of SNP strings needs to be calculated in the beginning of the clustering algorithm. This results in $C_{2}^{K}=O(K^{2})$computations. Later, to determine if two SNP-sets $C_{1}$ (containing $K_{1}$ SNPs) and $C_{2}$ (containing $K_{2}$ SNPs) should be merged, the Hamming distance between any $\mathbf{S}_{i}$ in $C_{1}$ and $\mathbf{S}_{j}$ in $C_{2}$ needs to be evaluated. This results in $O(K_{1}\times K_{2})$ computations. Fortunately, the latter calculation is not required because all dissimilarity metrics between any pair of SNPs have been evaluated in the first round of computation. Any subsequent procedure for evaluation of the distance $d(C_{i},C_{j})$ between any two SNP-sets $C_{i}$ and $C_{j}$ requires no further computation of Hamming distance. One only needs the id’s of the SNPs in both sets in order to retrieve the corresponding Hamming distance already derived in the first step. That is, our algorithm can expedite the speed of computation by using the same values and measurements throughout all procedures. This computational complexity is of less order than that of the k-mode and of Zhang’s method.

## Hamming Distance Association Test

If the Hamming distance association test (HDAT) is carried out on $N_{1}$ controls and $N_{2}$ cases, the computation complexity is $O(N_{m}^{2})$ where $N_{m}$ is the maximum of $N_{1}$ and $N_{2}$. If the SNP-set is of length $K$, then the order becomes $O(K\times N_{m}^{2})$. Again, the running time is not extensive. For instance, it took less than 30 seconds to carry out the HDAT for $K$=4 and $N_{1}=N_{2}$=500 with 1000 permutations in an ordinary desktop computer with Intel Core i7-4770 processor (3.40GHz) and 24GB RAM.
